# Interpretable Machine Learning Model Predicting Early Neurological Deterioration in Ischemic Stroke Patients Treated with Mechanical Thrombectomy: A Retrospective Study

**DOI:** 10.3390/brainsci13040557

**Published:** 2023-03-26

**Authors:** Tongtong Yang, Yixing Hu, Xiding Pan, Sheng Lou, Jianjun Zou, Qiwen Deng, Qingxiu Zhang, Junshan Zhou, Junrong Zhu

**Affiliations:** 1 Department of Pharmacy, Nanjing First Hospital, China Pharmaceutical University, Nanjing 210009, China; 2 Department of Neurology, Nanjing Hospital Affiliated to Nanjing Medical University, Nanjing 210009, China; 3 Department of Neurology, Drum Tower Hospital, Nanjing University, Nanjing 210009, China

**Keywords:** machine learning, acute ischemic stroke, early neurological deterioration, mechanical thrombectomy

## Abstract

Early neurologic deterioration (END) is a common and feared complication for acute ischemic stroke (AIS) patients treated with mechanical thrombectomy (MT). This study aimed to develop an interpretable machine learning (ML) model for individualized prediction to predict END in AIS patients treated with MT. The retrospective cohort of AIS patients who underwent MT was from two hospitals. ML methods applied include logistic regression (LR), random forest (RF), support vector machine (SVM), and extreme gradient boosting (XGBoost). The area under the receiver operating characteristic curve (AUC) was the main evaluation metric used. We also used Shapley Additive Explanation (SHAP) and Local Interpretable Model-Agnostic Explanations (LIME) to interpret the result of the prediction model. A total of 985 patients were enrolled in this study, and the development of END was noted in 157 patients (15.9%). Among the used models, XGBoost had the highest prediction power (AUC = 0.826, 95% CI 0.781–0.871). The Delong test and calibration curve indicated that XGBoost significantly surpassed those of the other models in prediction. In addition, the AUC in the validating set was 0.846, which showed a good performance of the XGBoost. The SHAP method revealed that blood glucose was the most important predictor variable. The constructed interpretable ML model can be used to predict the risk probability of END after MT in AIS patients. It may help clinical decision making in the perioperative period of AIS patients treated with MT.

## 1. Introduction

Recent clinical trials have shown that mechanical thrombectomy (MT) became the first-line standard treatment for acute ischemic stroke (AIS) patients caused by large vessel occlusion (LVO) [1,2,3,4,5]. However, AIS patients are susceptible to a common and feared complication of early neurologic deterioration (END) after MT [6], which is consistently associated with three-month unfavorable outcomes [7,8,9]. Therefore, it is desirable to predict the risk probability of END in AIS patients after MT and assist doctors in making more accurate treatment decisions for AIS patients.

Over the years, there have been many studies concerning exploring the predictors of END after MT in AIS patients, and some predictors have been found indeed, such as the blood glucose [10,11] baseline stroke severity [12], AIS patients with atrial fibrillation [13], diabetes mellitus [14,15], and so on. There are also several studies on predictive models for END after MT in AIS patients [16,17,18]. To some extent, these prediction models can help clinicians make more accurate treatment decisions for patients. However, these prediction models are based on traditional regression algorithms, which have certain limitations in solving nonlinear problems between various prognostic factors with outcomes. In addition, the variable selection based on the traditional statistical technique might obtain false positive variables or exclude false negative variables, which may debase predictive power. For overcoming the above problem, an alternative and effective technique is necessary for constructing precise prediction models.

Machine learning (ML) has emerged as a promising predictive tool in medicine and has been applied in many medical fields, such as ischemic stroke outcome prediction [19,20,21,22], biomedical research [23] and rehabilitation for chronic stroke survivors [24], and so on. Because in the process of modeling, machine learning can fit the complex relationship in multi-dimensional data, extract subtle information, and automatically summarize and generalize to obtain new knowledge. Machine learning has been limited in the medical field due to its black-box nature [25,26]. However, the latest machine learning models have been made interpretable by Shapley Additive Explanations (SHAP), which prompts the application of ML to be further developed [19,20]. The SHAP is a novel, cutting-edge machine learning algorithm which can visualize the relationship between each feature and the related predictive ability and can more intuitively understand the importance of features and enhance clinical interpretability.

We aimed to develop an interpretable machine learning model using patient preoperative and intraoperative relevant variables and demographics. The model was used to predict the probability of postoperative neurological deterioration and assist doctors to make more accurate treatment decisions for patients.

## 2. Methods

### 2.1. Study Population

This retrospective study was based on the clinical data of the patients with LVO who were treated with MT at the National Advanced Stroke Center of Nanjing First Hospital (China), from July 2015 to December 2021. We also used clinical data of the patients between January 2019 and December 2021 from Nanjing Drum Tower Hospital (China) for external validation to optimize the model. Inclusion criteria for this study can be listed as follows: (I) patients aged older than 18 years; (II) treated with MT; (III) anterior or posterior circulation large vessel occlusion state was verified by digital subtracted angiography, magnetic resonance angiography, or computed tomographic angiography. We excluded patients with uncertain 24 h neurological deterioration and lack of NIHSS score on admission. We also excluded patients with lacking vital data (i.e., age, sex and vascular recanalization status) and existing extreme outliers.

### 2.2. Patient Variables and Data Definitions

Patient information that was available for analyses included demographic characteristics, previous anti-thrombosis treatment, risk factors, past ischemic events, course data of stroke in 24 h, evaluation of the situation of stroke on admission with National Institutes of Health Stroke Scale [NIHSS] score, premorbid modified Rankin Scale [mRS] score, and laboratory findings (Table 1).

The symptomatic intracranial hemorrhage (sICH) happened within 24 h of admission, according to the Heidelberg Bleeding Classification [27]. Vascular recanalization was evaluated by modified Thrombolysis in Cerebral Infarction [mTICI] ≥ 2b [28]. The study was conducted in accordance with the Nanjing First Hospital, Nanjing Drum Tower Hospital, and approved by the document number of the ethics committee’s approval: ChiCTR-OCH-14004382. The outcome was END. END was defined as the NIHSS score with an increase of at least 4 points from baseline to 24 h of the stroke event [29].

### 2.3. Statistical Analysis

Categorical variables were expressed as numbers (percentages) and continuous variables were expressed as medians (quartile). Comparisons of the baseline characteristics between END and without END used the Student t-test or Mann–Whitney U test for continuous variables relying on the sample normality of distribution and Pearson’s test or Fisher’s exact test for categorical characteristics relying on the sample amount.

### 2.4. Data Processing and Feature Selection

Variables for which less than 20% of data were missing were included in the study, and missing variables were computed by using the median for continuous variables or the most common value for categorical variables, as the case may be. Before we started the modeling process, the dataset was split into training set (80%) and testing set (20%) with a stratified random sampling method. The data from Nanjing Drum Tower Hospital was taken as an external validation set. The training set was applied to the step of feature selection and training models. To overcome the imbalance of the training set, we applied stratified random sampling to generate positive cases, which may effectively prevent the overfitting problem. The testing set was used for evaluating model performance during and after training, while the validation set was used for evaluating the generalization of the model.

Redundant and extraneous factors would draw too much detail and noise, which may be prone to form a model over-fitting and decrease the predictive ability of the model, respectively. Therefore, we utilized the Least Absolute Selection and Shrinkage Operator (LASSO) [30] to exclude non-interference variables and developed ML models. It has the advantage of combining packaging method with machine learning algorithm and also has the advantage of high computational efficiency of filtering method. In brief, the LASSO is a regularized regression technique commonly used for reducing a high-dimensional feature space. Its principle is to shrink some coefficients to exactly zero and thus perform variable selection.

### 2.5. Modeling Strategies

**Figure 1:**
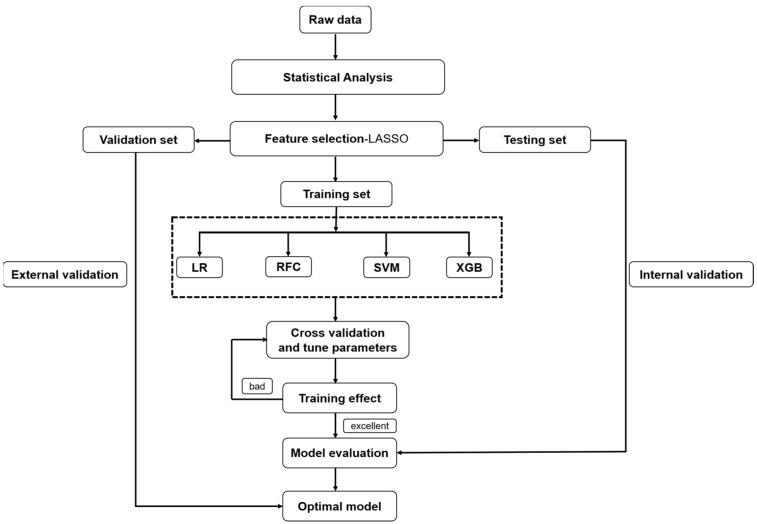
Modeling procedure. Machine learning involved feature selection by LASSO, model building with five algorithms. LASSO, Least Absolute Selection and Shrinkage Operator; LR, Logistic Regression; RF, Random Forest; SVM, Support Vector Machine; XGBoost, Extreme Gradient Boosting.

Modeling processes were shown in Figure 1. The END ML model was built with data from the training set. We initially attempted to use four ML algorithms for constructing models, and these four models were named logistic regression (LR), random forest (RF), extreme gradient boosting (XGBoost), and support vector machine (SVM). We used 10-fold cross-validation technique to improve generality, and grid search algorithm was used for tuning hyperparameters for each model.

### 2.6. Model Evaluation

Model performance was assessed by area under the receiver operating characteristic curve (AUC) using the independent testing set. The Delong test and calibration curves were used for comparing the ROC curves in different models, which could identify the optimal model. Calibration curves of the models were evaluated by using the Brier score method (range: 0–1), with lower scores reflecting better model calibration, which showed the agreement between the model’s predicted values and the cohort’s observed outcome [31]. We also input the validation set to assess the generalization of model. The sensitivity, specificity, accuracy (ACC), and Youden index were also analyzed.

### 2.7. Explanation of the Model

Although the above algorithms are robust and well-performing ML methods that have been very popular in the medical field, it is difficult to interpret and display black-box characters. Thus, based on the training set, we introduced the SHAP to our prediction model to make up for the issue of black-box character, which can obtain analyses of the features and make patient-specific predictions rationally [32]. The SHAP was inspired by cooperative game theory, which could visually represent the importance ranking of features and calculate each feature Shapley values in the prediction model [33]. In addition, to achieve a better interpretation of the prediction model in individual patients, we also introduced Local Interpretable Model-Agnostic Explanations (LIME) to exhibit the impact of vital variables at the level of the individual. Briefly, based on a local linear model, LIME represents a detailed interpretation of a classifier by approximating weights to the disturbance input. Thus, we used LIME to explain two specific instances in the best predictive behavior of the model. Python version 3.7 was used in this present study and related packages include XGBoost, Shap, and Scikit-learn environment.

## 3. Results

### 3.1. Study Population

Table 1 describes the characteristics of the study population from Nanjing First Hospital. A total of 985 AIS patients with LVO and 36 features were taken into the study, comprising 157 who developed END during hospitalization and 828 without END. The univariate analysis revealed that coronary artery disease, sICH, Stent retriever only, Stent retriever/aspiration with rescue therapy, blood glucose, and glycated hemoglobin were associated with the risk of END. Supplementary Table S1 describes the demographics and clinical characteristics of the external validation set: there are 56 positive cases and 177 negative cases.

### 3.2. Feature Selection

The data set was randomly split into the training set (*n* = 690) and the testing set (*n* = 295). In the LASSO algorithm, 9 variables, namely, blood glucose, NIHSS at baseline, interval from groin puncture to recanalization, serum creatinine, interval from onset to treatment, systolic blood pressure, diastolic blood pressure, platelets, and uric acid were selected as crucial variables, which were determined by the result of LASSO regularization process based on 690 patients in the training set.

### 3.3. Model Building and Evaluation

**Figure 2:**
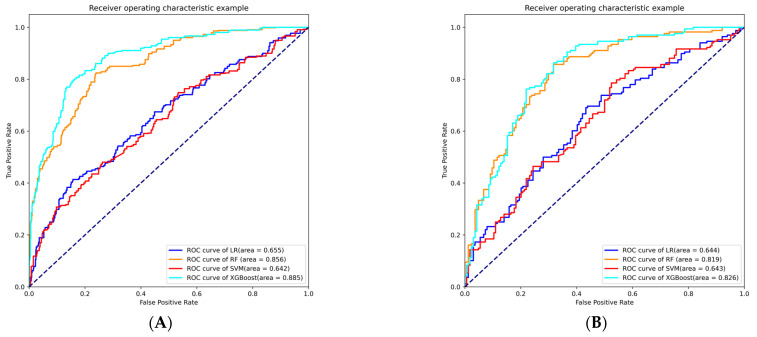
The receiver operating characteristic curve (ROC) of five models on training set (**A**) and ROC of five models on testing set (**B**). LR, logistic regression; RF, random forest; SVM, support vector machine; XGBoost, extreme gradient boosting.

**Figure 3:**
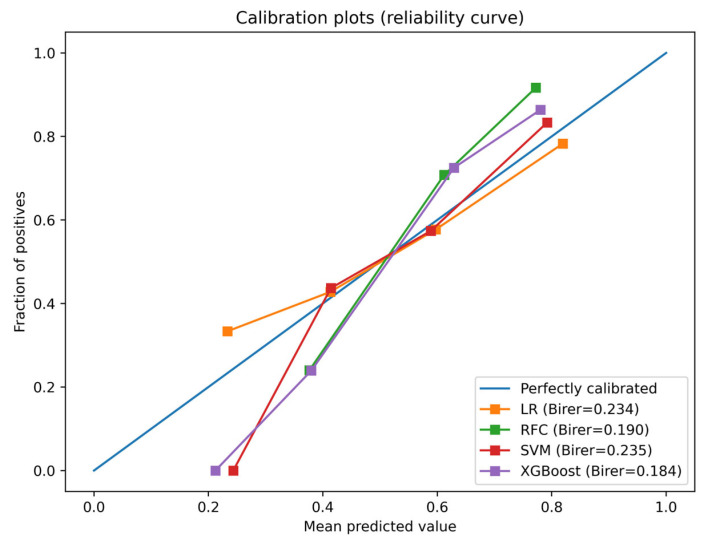
The calibration curves and the Brier score of machine learning models on the testing set of models. LR, logistic regression; RF, random forest; SVM, support vector machine; XGBoost, extreme gradient boosting.

We applied the following ML algorithm with 9 crucial variables as input variables, including LR, XGBoost, SVM, RFC, and DNN to predict the risk of END. Figure 2 shows the ROC of each model on the training set and testing set. Table 2 shows the evaluation indexes of each model on the testing set, including AUC, sensitivity, specificity, accuracy, and the Youden index. Overall, among the four models, XGBoost had the highest prediction power (AUC = 0.826, 95% CI 0.781–0.871), whereas SVM showed the poorest prediction performance (AUC = 0.643, 95% CI 0.584–0.702). The Delong test was used for comparing the performance of the 4 models on the testing set, and *p* < 0.05 were considered statistically significant. Supplementary Table S2 shows that RF and XGBoost significantly surpassed those of the other models in prediction. We further determined the optimal model from the calibration curve; the plot showed that the XGBoost had the lowest brier score, which means the model has the best calibration (Figure 3). Therefore, the XGBoost was selected to be the optimal model.

As shown in Table 3, we validated the XGBoost model in an external validation cohort with 233 patients, and the AUC in the validating set was 0.846. We further calculated the confusion matrix, the sensitivity, specificity, and overall accuracy were 0.750, 0.836, and 0.815, which means that among the 56 cases with END, 46 cases were correctly predicted by the model, while among the 177 cases without END, 148 cases were correctly predicted by the model.

### 3.4. Explanation of the Model at the Feature Level

**Figure 4:**
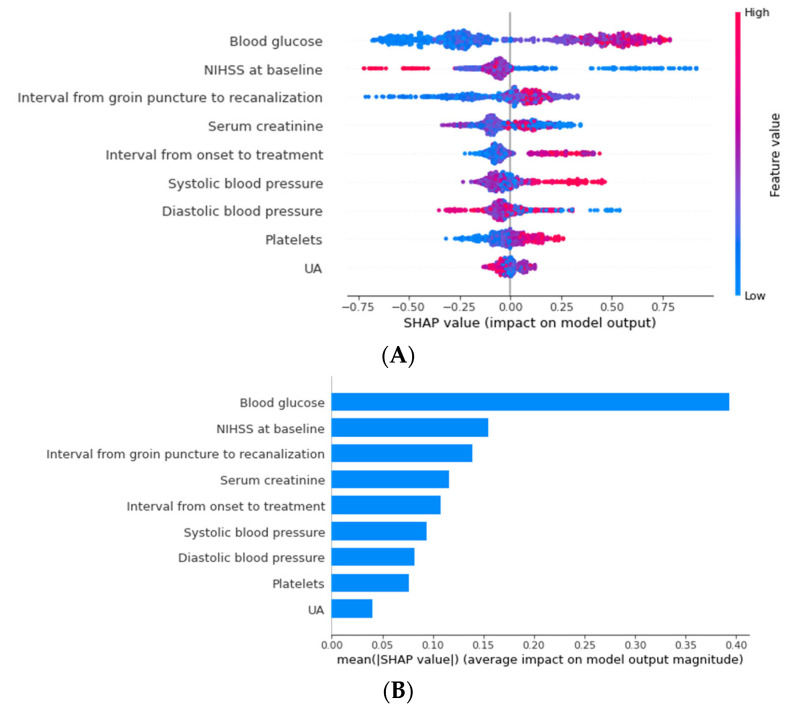
Feature importance ranking based on Shapley Additive Explanation (SHAP) values. Red indicates that the value of the feature is high, and blue indicates that the value of the feature is low; the x-axis represents the SHAP values; the features are ranked according to the sum of the SHAP values for all patients (**A**). Standard bar charts were drawn and sorted using the average absolute value of the shape values of each feature in model (**B**).

The SHAP algorithm has been applied to the XGBoost model to obtain each variable’s importance for the END prediction. The importance distribution for each variable in descending order was plotted in Figure 4A. Blood glucose was the strongest predictor of END, followed closely by NIHSS at baseline, interval from groin puncture to recanalization, serum creatinine, and interval from onset to treatment. In addition, in order to distinguish the relationship between the target result and positive and negative predictors, SHAP values were used for uncovering the END risk factors. As shown in Figure 4B, each row represents a feature, and each dot represents a sample; the redder the color, the greater the value of the feature; the bluer the color, the smaller the value of the feature. What can be found is that increases in the concentration of blood glucose are a positive impact and are more likely to develop END, whereas increases in NIHSS at baseline have a negative impact and is less likely to develop END.

### 3.5. Explanation of the Model at the Individual Level

**Figure 5:**
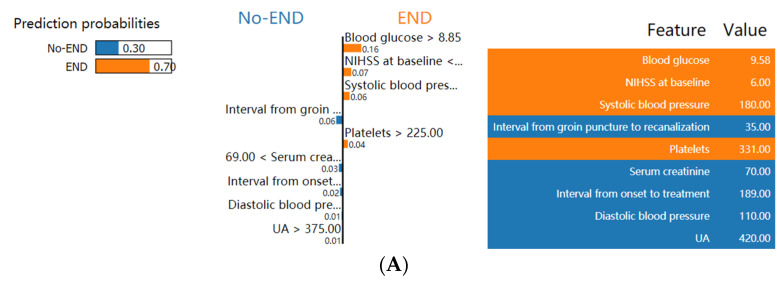

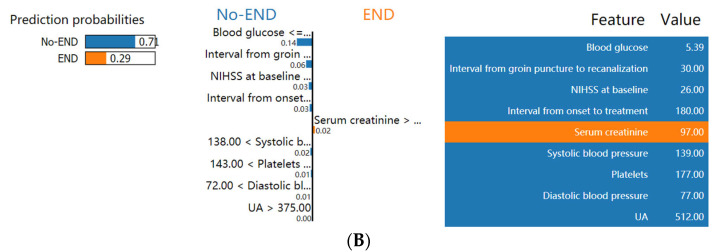
LIME plot for individual case explanation on two random patients from the testing set of the XGBoost model. LIME plot included the patient from the “true positive” group explained by LIME algorithm (**A**) and a patient from the “true negative” group explained by LIME algorithm (**B**).

We next used LIME to analyze the contribution level of features of new instances for the prediction of END in LVO patients. As illustrated in Figure 5, the true values of the nine main features (right), the overall predicted probability of END and No-END (left), and the classification details (middle) of the two instances were exhibited in the LIME plot. For instance, in patient 23, the predicted probability for END was high (0.70) due to the number of positive conditions, consisting of a high concentration of blood glucose, low NIHSS at baseline, high systolic blood pressure and high platelets. In contrast, the END probability in patient 100 was low (0.29) due to few positive conditions, only high serum creatinine.

## 4. Discussion

As far as we know, this study is the first attempt to use the explainable machine learning model to predict the risk of END after MT for AIS patients. Moreover, the results of the Delong test showed that our model had good discrimination. Additionally, the prediction accuracy of our model was high. Among the 4 ML prediction models, XGBoost had the best prediction effect, with an AUC as high as 0.826. The XGBoost algorithm can control the complexity of the model by adding regular terms, which is more conducive to preventing overfitting. Furthermore, it can better deal with classified and multi-dimensional data sets and improve the computational power and generalization ability of the model, which makes it suitable for clinical application. We also used the data of Nanjing Drum Tower Hospital to externally validate our prediction model, and the AUC value was as high as 0.846, indicating that our model has a high potential for prediction of END in a wider range of the Chinese population at least. We hope that more data can be used to further validate our model in the future. In our prediction model, some variables associated with END were not previously highlighted, such as the interval from onset to treatment, platelet, uric acid, and so on. In our interpretable ML model, the variables most associated with END were visualized, which helps to more intuitively understand the risk factors associated with END.

Our study is consistent with previous findings and validates the value of the ML model in predicting END after MT in AIS patients. The fasting blood glucose level [34,35,36,37] and the baseline NIHSS score [35,37] have been reported as risk factors for END. These risk factors have been reported in previous studies. Following previous work has shown that it may be related to vascular endothelial dysfunction caused by impaired blood glucose control [38]. Recent studies have shown that there is a close link between pre-existing hyperglycemia and increased cerebral ischemia/reperfusion injury in the field of stroke. In addition, previous studies have shown that clinical features alone that are observed at stroke onset can help to distinguish cardioembolic from atherothrombotic infarction [39]. However, in our study, although the incidence of END in patients with atherosclerotic infarcts is higher than that in patients with cardioembolic stroke, this is not a statistically significant difference (*p* > 0.05). This may require us to expand the sample size and do further research in the future.

The thromboinflammation will be further developed mainly due to the high blood glucose, which creates a harmful environment in the body [40]. The study by Girot et al. in the field of END showed that a low NIHSS score on admission was an independent risk factor [17]. Our findings are consistent with Girot et al. At first glance, this result may be surprising. It is possible that those with higher NIHSS scores are less prone to increase their score compared to those with lower NIHSS scores. It may be that patients with lower NIHSS scores are prone to vascular injuries in the acute phase and hypoperfusion areas that lead to infarction progression. Rohini et al. [18] showed that higher SBP is highly associated with END, which is consistent with our findings. It could be explained that patients with high SBP may have more severe strokes. In addition, previous studies have shown that elevated baseline SBP in AIS patients may be associated with death and dependence [41]. In our study, the time from groin puncture to revascularization and the time from symptom onset to treatment were also highly correlated with END. The time from onset to revascularization was an independent predictor of END after EVT for acute basilar artery occlusion, which was reported by Zhong et al. [42].

The complexity of ML models makes it difficult to determine the reasons behind their predictions, which may hinder clinical application. However, in this study, the SHAP algorithm was used to interpret the prediction at different levels, which ensured the performance and clinical interpretability of the model and was demonstrated to the user through friendly visualization tools. Clinicians will have a better understanding of the decision-making process of the model, which is conducive to the clinical use of the prediction results. Furthermore, it is demonstrated in our study that interpretable machine learning methods are capable of predicting END and individualizing predictions in the context of patients. Previous studies on END mainly focused on its pathophysiological explanation and each risk factor, which lacked the combined usage of large samples in clinical practice [43,44,45]. Furthermore, there is no uniformly accepted risk stratification algorithm for predicting END. Therefore, the strength of our ML model is that relevant variables can be extracted from real-world clinical data to predict END.

Our study has some limitations. First, although we have data for external validation, this is retrospective data, and the data comes from one center. The retrospective data might have led to recall, and selection bias to a certain degree. Therefore, in order to promote the model, more data sets and prospective multicenter clinical trials are needed to verify our results and the accuracy of the model. Second, to retain more available data, we only considered medical records in which END occurred within 24 h after stroke, although END is also known to occur within days of the initial time. In addition, some anesthetic drugs were not completely metabolized in the patient, and the patient was still intubated, which would lead to a risk of bias in the NIHSS score. Third, the collateral status may affect END, but this variable was not collected. In addition, in the process of building the model, feature selection was only applied once on the combined training validation set without being incorporated into the cross-validation splits. Therefore, it is possible that some feature importance variabilities were unaccounted for. Finally, the participants are all from the Chinese population, so the results cannot be easily extrapolated to other groups.

## 5. Conclusions

In this study, the interpretable ML model constructed can be used to predict the risk probability of END after MT in AIS patients. In addition, the model was externally validated and achieved good predictive performance. However, the amount of data to validate the model is small, and more clinical data and prospective clinical studies are needed for further verification. Furthermore, the results from this study may guide clinical decision making in the selection and intraoperative and postoperative management of AIS patients who had undergone MT with a high risk of END.

## Figures and Tables

**Table 1:** Demographics and clinical characteristics according to patients with and without early neurological deterioration.

Variables	All Patients, *n* = 985	with END, *n* = 157	without END, *n* = 828	*p* Value
**Demographic characteristics**				
Age, years, median (IQR)	71.0(63.0–79.0)	71.0(63.5–80.0)	71.0(63.0–79.0)	0.620
Female, *n* (%)	377(38.3)	61(38.9)	316(38.2)	0.871
**Vascular risk factors, *n* (%)**				
Hypertension	677(68.7)	117(74.5)	560(67.6)	0.088
Diabetes mellitus	232(23.6)	46(29.3)	186(22.5)	0.064
Hyperlipidemia	17(1.7)	3(1.9)	14(1.7)	0.846
Coronary artery disease	142(14.4)	13(8.3)	129(15.6)	0.017
Atrial fibrillation	311(31.6)	43(27.4)	268(32.4)	0.219
Previous stroke or TIA	215(21.8)	30(19.1)	185(22.3)	0.368
Smoking	303(30.8)	45(28.7)	258(31.2)	0.524
Drinking	232(23.6)	28(17.8)	204(24.6)	0.065
**Clinical data, median (IQR)**				
Systolic blood pressure, mmHg	138.0(123.0–155.0)	140.0(125.0–160.0)	138.0(123.0–154.0)	0.196
Diastolic blood pressure, mmHg	82.0(73.0–93.0)	82.0(71.5–93.5)	82.5(73.0–93.0)	0.991
NIHSS at baseline	14.0(11.0–18.0)	14.0(8.5–18.0)	14.0(11.0–18.0)	0.186
Interval from onset to treatment, min	270.0(190.0–410.0)	300.0(189.5–500.0)	270.0(190.0–404.3)	0.426
Interval from groin puncture to recanalization, min	70.0(50.0–95.0)	72.0(55.0–104.5)	68.0(50.0–94.3)	0.089
**Cause of stroke, *n* (%)**				0.067
Atherosclerotic	448(45.5)	76(48.4)	372(44.9)	
Cardioembolic	433(44)	58(36.9)	375(45.3)	
Others	104(10.6)	23(14.6)	81(9.8)	
**Endovascular therapy, *n* (%)**				
Intravenous thrombolysis	379(38.5)	64(40.8)	315(38)	0.521
Tirofiban	425(43.1)	74(47.1)	351(42.4)	0.271
sICH	139(14.1)	39(24.8)	100(12.1)	0.000
Recanalization	883(89.6)	134(85.4)	749(90.5)	0.054
**Lesion location, *n* (%)**				
Anterior circulation	790(80.2)	117(74.5)	673(81.3)	0.051
Posterior circulation	197(20)	40(25.5)	157(19)	0.061
**Procedural modes**				
Aspiration only, *n* (%)	34(3.5)	8(5.1)	26(3.1)	0.218
Stent retriever only, *n* (%)	740(75.1)	104(66.2)	636(76.8)	0.005
Stent retriever/aspiration with rescue therapy, *n* (%)	228(23.1)	47(29.9)	181(21.9)	0.028
Passes of Stent retriever, median (IQR)	2.0(1.0–3.0)	2.0(1.0–3.0)	2.0(1.0–3.0)	0.830
**Laboratory data, median (IQR)**				
Platelets, μmol/L	180.0(145.0–220.0)	185.0(145.5–228.5)	180.0(145.0–219.0)	0.433
Serum creatinine, μmol/L	69.0(58.0–83.5)	69.7(55.8–90.5)	69.0(58.0–83.0)	0.825
Blood glucose, mmol/L	6.6(5.4–8.2)	7.9(6.1–9.8)	6.4(5.3–7.8)	0.000
Total cholesterol, mmol/L	4.1(3.4–5.0)	4.1(3.5–5.0)	4.1(3.4–4.9)	0.476
Triglyceride, mmol/L	1.0(0.8–1.5)	1.1(0.8–1.5)	1.0(0.7–1.4)	0.074
High-density lipoprotein, mmol/L	1.1(0.9–1.3)	1.1(0.9–1.3)	1.1(0.9–1.3)	0.391
Low-density lipoprotein, mmol/L	2.5(1.9–3.1)	2.4(1.8–3.2)	2.5(1.9–3.1)	0.973
UA, μmol/L	314.0(241.5–383.0)	321.0(246.0–382.5)	313.0(241.0–383.0)	0.604
Glycated hemoglobin, mmol/L	5.9(5.6–6.6)	6.2(5.7–6.8)	5.9(5.6–6.5)	0.004
Homocysteine, μmol/L	13.1(10.9–15.7)	13.1(10.4–15.7)	13.1(11.0–15.7)	0.697

Data are presented as median (IQR) or number (%). Abbreviations: IQR, interquartile range; TIA, transient cerebral ischemia; NIHSS, National Institute of Health stroke scale; sICH, symptomatic intracranial hemorrhage; UA, uric acid.

**Table 2:** Discrimination and calibration of each machine learning algorithm on the testing set.

Model	AUC	Sensitivity	Specificity	Accuracy	Brier	Youden Index
LR	0.644	0.387	0.793	0.587	0.234	0.565
RF	0.819	0.827	0.689	0.759	0.190	0.480
SVM	0.643	0.482	0.713	0.596	0.235	0.520
XGBoost	0.826	0.798	0.713	0.756	0.184	0.509

AUC, area under the curve; LR, logistic regression; RF, random forest; SVM, support vector machine; XGBoost, extreme gradient boosting.

**Table 3:** Prediction performance for the external validation cohort.

Model	AUC	Sensitivity	Specificity	Accuracy
XGBoost	0.846	0.750	0.836	0.815

AUC, area under the curve; XGBoost, extreme gradient boosting.

## Data Availability

The datasets used in this study are available from the corresponding author upon reasonable request.

## Supplementary Materials

The following supporting information can be downloaded at: https://www.mdpi.com/article/10.3390/brainsci13040557/s1, Table S1: Demographics and clinical characteristics of external validation set; Table S2: The *p*-values of pairwise comparisons of AUCs on the testing set for different models with the Delong test.

## References

[1] Saver J.L., Goyal M., Bonafe A., Diener H.C., Levy E.I., Pereira V.M., Albers G.W., Cognard C., Cohen D.J., Hacke W. (2015). Stent-retriever thrombectomy after intravenous t-PA vs. t-PA alone in stroke. N. Engl. J. Med..

[2] Powers W.J., Rabinstein A.A., Ackerson T., Adeoye O.M., Bambakidis N.C., Becker K., Biller J., Brown M., Demaerschalk B.M., Hoh B. (2019). Guidelines for the Early Management of Patients With Acute Ischemic Stroke: 2019 Update to the 2018 Guidelines for the Early Management of Acute Ischemic Stroke: A Guideline for Healthcare Professionals From the American Heart Association/American Stroke Association. Stroke.

[3] Goyal M., Demchuk A.M., Menon B.K., Eesa M., Rempel J.L., Thornton J., Roy D., Jovin T.G., Willinsky R.A., Sapkota B.L. (2015). Randomized assessment of rapid endovascular treatment of ischemic stroke. N. Engl. J. Med..

[4] Campbell B.C., Mitchell P.J., Kleinig T.J., Dewey H.M., Churilov L., Yassi N., Yan B., Dowling R.J., Parsons M.W., Oxley T.J. (2015). Endovascular therapy for ischemic stroke with perfusion-imaging selection. N. Engl. J. Med..

[5] Berkhemer O.A., Fransen P.S., Beumer D., van den Berg L.A., Lingsma H.F., Yoo A.J., Schonewille W.J., Vos J.A., Nederkoorn P.J., Wermer M.J. (2015). A randomized trial of intraarterial treatment for acute ischemic stroke. N. Engl. J. Med..

[6] Saver J.L., Altman H. (2012). Relationship between neurologic deficit severity and final functional outcome shifts and strengthens during first hours after onset. Stroke.

[7] Mori M., Naganuma M., Okada Y., Hasegawa Y., Shiokawa Y., Nakagawara J., Furui E., Kimura K., Yamagami H., Kario K. (2012). Early neurological deterioration within 24 hours after intravenous rt-PA therapy for stroke patients: The Stroke Acute Management with Urgent Risk Factor Assessment and Improvement rt-PA Registry. Cerebrovasc. Dis..

[8] Dharmasaroja P.A., Muengtaweepongsa S., Dharmasaroja P. (2011). Early outcome after intravenous thrombolysis in patients with acute ischemic stroke. Neurol. India.

[9] Davalos A., Toni D., Iweins F., Lesaffre E., Bastianello S., Castillo J. (1999). Neurological deterioration in acute ischemic stroke: Potential predictors and associated factors in the European cooperative acute stroke study (ECASS) I. Stroke.

[10] Simonsen C.Z., Schmitz M.L., Madsen M.H., Mikkelsen I.K., Chandra R.V., Leslie-Mazwi T., Andersen G. (2016). Early neurological deterioration after thrombolysis: Clinical and imaging predictors. Int. J. Stroke.

[11] Bourcier R., Goyal M., Muir K.W., Desal H., Dippel D.W.J., Majoie C., van Zwam W.H., Jovin T.G., Mitchell P.J., Demchuk A.M. (2022). Risk factors of unexplained early neurological deterioration after treatment for ischemic stroke due to large vessel occlusion: A post hoc analysis of the HERMES study. J. Neurointerv. Surg..

[12] Haeusler K.G., Gerischer L.M., Vatankhah B., Audebert H.J., Nolte C.H. (2011). Impact of hospital admission during nonworking hours on patient outcomes after thrombolysis for stroke. Stroke.

[13] Gong P., Zhang X., Gong Y., Liu Y., Wang S., Li Z., Chen W., Zhou F., Zhou J., Jiang T. (2020). A novel nomogram to predict early neurological deterioration in patients with acute ischaemic stroke. Eur. J. Neurol..

[14] Tanaka R., Ueno Y., Miyamoto N., Yamashiro K., Tanaka Y., Shimura H., Hattori N., Urabe T. (2013). Impact of diabetes and prediabetes on the short-term prognosis in patients with acute ischemic stroke. J. Neurol. Sci..

[15] Kim B.J., Park J.M., Kang K., Lee S.J., Ko Y., Kim J.G., Cha J.K., Kim D.H., Nah H.W., Han M.K. (2015). ERRATUM: Table Correction: Case Characteristics, Hyperacute Treatment, and Outcome Information from the Clinical Research Center for Stroke-Fifth Division Registry in South Korea. J. Stroke.

[16] Sun D., Tong X., Huo X., Jia B., Raynald, Wang A., Ma G., Ma N., Gao F., Mo D. (2022). Unexplained early neurological deterioration after endovascular treatment for acute large vessel occlusion: Incidence, predictors, and clinical impact: Data from ANGEL-ACT registry. J. Neurointerv. Surg..

[17] Girot J.B., Richard S., Gariel F., Sibon I., Labreuche J., Kyheng M., Gory B., Dargazanli C., Maier B., Consoli A. (2020). Predictors of Unexplained Early Neurological Deterioration After Endovascular Treatment for Acute Ischemic Stroke. Stroke.

[18] Bhole R., Nouer S.S., Tolley E.A., Turk A., Siddiqui A.H., Alexandrov A.V., Arthur A.S., Mocco J., COMPASS Investigators (2022). Predictors of early neurologic deterioration (END) following stroke thrombectomy. J. Neurointerv. Surg..

[19] Yao Z., Mao C., Ke Z., Xu Y. (2022). An explainable machine learning model for predicting the outcome of ischemic stroke after mechanical thrombectomy. J. Neurointerv. Surg..

[20] Jabal M.S., Joly O., Kallmes D., Harston G., Rabinstein A., Huynh T., Brinjikji W. (2022). Interpretable Machine Learning Modeling for Ischemic Stroke Outcome Prediction. Front. Neurol..

[21] Mistry E.A., Yeatts S., de Havenon A., Mehta T., Arora N., De Los Rios La Rosa F., Starosciak A.K., Siegler J.E., Mistry A.M., Yaghi S. (2021). Predicting 90-Day Outcome After Thrombectomy: Baseline-Adjusted 24-Hour NIHSS Is More Powerful Than NIHSS Score Change. Stroke.

[22] Hu Y., Yang T., Zhang J., Wang X., Cui X., Chen N., Zhou J., Jiang F., Zhu J., Zou J. (2022). Dynamic Prediction of Mechanical Thrombectomy Outcome for Acute Ischemic Stroke Patients Using Machine Learning. Brain Sci..

[23] Zorman M., Verlic M. (2009). Explanatory approach for evaluation of machine learning-induced knowledge. J. Int. Med. Res..

[24] Chae S.H., Kim Y., Lee K.S., Park H.S. (2020). Development and Clinical Evaluation of a Web-Based Upper Limb Home Rehabilitation System Using a Smartwatch and Machine Learning Model for Chronic Stroke Survivors: Prospective Comparative Study. JMIR Mhealth Uhealth.

[25] Yu M.K., Ma J., Fisher J., Kreisberg J.F., Raphael B.J., Ideker T. (2018). Visible Machine Learning for Biomedicine. Cell.

[26] Tjoa E., Guan C. (2021). A Survey on Explainable Artificial Intelligence (XAI): Toward Medical XAI. IEEE Trans. Neural. Netw. Learn Syst..

[27] von Kummer R., Broderick J.P., Campbell B.C., Demchuk A., Goyal M., Hill M.D., Treurniet K.M., Majoie C.B., Marquering H.A., Mazya M.V. (2015). The Heidelberg Bleeding Classification: Classification of Bleeding Events After Ischemic Stroke and Reperfusion Therapy. Stroke.

[28] Zhang X., Yuan K., Wang H., Gong P., Jiang T., Xie Y., Sheng L., Liu D., Liu X., Xu G. (2020). Nomogram to Predict Mortality of Endovascular Thrombectomy for Ischemic Stroke Despite Successful Recanalization. J. Am. Heart Assoc..

[29] Siegler J.E., Boehme A.K., Kumar A.D., Gillette M.A., Albright K.C., Martin-Schild S. (2013). What change in the National Institutes of Health Stroke Scale should define neurologic deterioration in acute ischemic stroke?. J. Stroke Cerebrovasc. Dis..

[30] Tibshirani R. (2011). Regression shrinkage and selection via the lasso: A retrospective. J. R. Stat. Soc. Ser. B-Stat. Methodol..

[31] Jelovsek J.E., Hill A.J., Chagin K.M., Kattan M.W., Barber M.D. (2016). Predicting Risk of Urinary Incontinence and Adverse Events After Midurethral Sling Surgery in Women. Obstet. Gynecol..

[32] Rodriguez-Perez R., Bajorath J. (2020). Interpretation of Compound Activity Predictions from Complex Machine Learning Models Using Local Approximations and Shapley Values. J. Med. Chem..

[33] Thorsen-Meyer H.C., Nielsen A.B., Nielsen A.P., Kaas-Hansen B.S., Toft P., Schierbeck J., Strom T., Chmura P.J., Heimann M., Dybdahl L. (2020). Dynamic and explainable machine learning prediction of mortality in patients in the intensive care unit: A retrospective study of high-frequency data in electronic patient records. Lancet Digit. Health.

[34] Yu Q., Mao X., Fu Z., Luo S., Huang Q., Chen Q., Li S., Zhang J., Qiu Y., Wu Y. (2022). Fasting blood glucose as a predictor of progressive infarction in men with acute ischemic stroke. J. Int. Med. Res..

[35] Seners P., Turc G., Oppenheim C., Baron J.C. (2015). Incidence, causes and predictors of neurological deterioration occurring within 24 h following acute ischaemic stroke: A systematic review with pathophysiological implications. J. Neurol. Neurosurg. Psychiatry.

[36] Kim J.S., Kim R.Y., Cha J.K., Rha H.W., Kang M.J., Kim D.H., Park H.S., Choi J.H., Huh J.T., Lee I.K. (2017). Pre-stroke glycemic control is associated with early neurologic deterioration in acute atrial fibrillation-related ischemic stroke. eNeurologicalSci.

[37] Duan Z., Guo W., Tang T., Tao L., Gong K., Zhang X. (2020). Relationship between high-sensitivity C-reactive protein and early neurological deterioration in stroke patients with and without atrial fibrillation. Heart Lung.

[38] Jamwal S., Sharma S. (2018). Vascular endothelium dysfunction: A conservative target in metabolic disorders. Inflamm. Res..

[39] Arboix A., Oliveres M., Massons J., Pujades R., Garcia-Eroles L. (1999). Early differentiation of cardioembolic from atherothrombotic cerebral infarction: A multivariate analysis. Eur. J. Neurol..

[40] Desilles J.P., Syvannarath V., Ollivier V., Journe C., Delbosc S., Ducroux C., Boisseau W., Louedec L., Di Meglio L., Loyau S. (2017). Exacerbation of Thromboinflammation by Hyperglycemia Precipitates Cerebral Infarct Growth and Hemorrhagic Transformation. Stroke.

[41] Petersen N.H., Ortega-Gutierrez S., Wang A., Lopez G.V., Strander S., Kodali S., Silverman A., Zheng-Lin B., Dandapat S., Sansing L.H. (2019). Decreases in Blood Pressure During Thrombectomy Are Associated with Larger Infarct Volumes and Worse Functional Outcome. Stroke.

[42] Zhong X., Tong X., Sun X., Gao F., Mo D., Wang Y., Miao Z. (2020). Early Neurological Deterioration Despite Recanalization in Basilar Artery Occlusion Treated by Endovascular Therapy. Front. Neurol..

[43] Sun W., Liu W., Zhang Z., Xiao L., Duan Z., Liu D., Xiong Y., Zhu W., Lu G., Liu X. (2014). Asymmetrical cortical vessel sign on susceptibility-weighted imaging: A novel imaging marker for early neurological deterioration and unfavorable prognosis. Eur. J. Neurol..

[44] Seo W.K., Seok H.Y., Kim J.H., Park M.H., Yu S.W., Oh K., Koh S.B., Park K.W. (2012). C-reactive protein is a predictor of early neurologic deterioration in acute ischemic stroke. J. Stroke Cerebrovasc. Dis..

[45] Kwon H.M., Lee Y.S., Bae H.J., Kang D.W. (2014). Homocysteine as a predictor of early neurological deterioration in acute ischemic stroke. Stroke.

