# Supplementary material for: Interpretable Machine Learning Model Predicting Early Neurological Deterioration in Ischemic Stroke Patients Treated with Mechanical Thrombectomy: A Retrospective Study
Source: Brain Sci. 2023 Mar 26;13(4):557. doi: 10.3390/brainsci13040557 (PMC10137089; doi:10.3390/brainsci13040557)
Supplement: Supplementary file 1 [file brainsci-13-00557-s001.zip › brainsci-2273257-supplementary.pdf]

Supplement Table S1. Demographics and clinical characteristics of external validation set

Vriables	All patients, n = 233	With END, n = 56	Without END, n = 177
**Demographic characteristics**			
Age, years, median (IQR)	71.0(62.0-80.0)	72.5(65.3-81.8)	70.0(61.5-78.5)
Female, n (%)	103(44.2)	26 (46.4)	77(43.5)
**Vascular risk facors, n (%)**			
Hypertension	156(66.9)	43(76.8)	113(63.8)
Diabetes mellitus	61(26.1)	18(32.1)	43 (24.3)
Hyperlipidemia	7(3.0)	3(5.4)	4(2.3)
Coronary artery disease	25(10.7)	7(10.2)	18(12.5)
Atrial fibrillation	87(37.3)	20(35.7)	67(37.9)
Previous stroke or TIA	38(16.3)	10(17.9)	28(15.8)
Smoking, n (%)	50(21.4)	12(21.4)	38(21.5)
Drinking, n (%)	38(16.3)	9(16.1)	29(16.4)
**Clinical data**			
Systolic blood pressure, mmHg, median (IQR)	139.5(123.5-160.0)	141.0(127.0-169.8)	139.0(123.0-159.5)
Diastolic blood pressure, mmHg, median (IQR)	79.0(68.5-90.0)	80.7(70.0-96.0)	78.0(67.0-90.0)
NIHSS at baseline, score, median (IQR)	15.0(11.0-18.5)	16.0(12.0-19.5)	14.0(11.0-18.5)
Interval from onset to treatment, median (IQR)	355.0(270.0-500.0)	357.5(270.0-555.0)	355.0(260.0-476.5)
Interval from groin puncture to recanalization, min, median (IQR)	65.0(38.5-105.0)	76.5(45.3-137.5)	60.0(37.0-100.0)
**Cause of stroke, n (%)**			
Atherosclerotic	93(39.9)	22(39.3)	71 (40.1)
Cardioembolic	107(45.9)	27(48.2)	80(45.2)
Others	33(14.2)	7(12.5)	26(14.7)
Intravenous thrombolysis, n (%)	62(26.6)	13(23.2)	49(27.7)
**Endovascular therapy**			
Tirofiban, n (%)	179(76.8)	41(73.2)	138(78.0)
sICH, n (%)	65(27.8)	17(30.4)	48(27.1)
Recanalization, n (%)	215(92.2)	46(82.1)	169(95.5)
**Lesion location, n (%)**			
Anterior circulation	145(62.2)	29(51.8)	116(65.5)
Posterior circulation	88(37.7)	27(48.2)	61(34.5)
**Procedural modes, n (%)**			
Aspiration only	12(5.1)	1(1.8)	11 (6.2)
Stent retriever only	153(65.6)	35(62.5)	118(66.7)
Stent retriever/aspiration with rescue therapy	68(29.1)	20(35.7)	48(27.1)
Passes of stent retriever, median (IQR)	2.0(1.0-3.0)	2.0(1.0-3.0)	2.0(1.0-3.0)
**Laboratory data, median (IQR)**			
Platelets, μmol/L	178.0(151.0-213.0)	176.0(146.8-226.0)	178.0(152.0-211.0)
Serum creatinine, μmol/L	65.0(55.0-78.5)	68.5(54.0-82.8)	65.0(56.0-76.0)
Blood glucose, mmol/L	6.7(5.4-8.8)	7.9(6.0-11.0)	6.4(5.3-7.6)
Total cholesterol, mmol/L	4.1(3.5-5.0)	4.1(3.4-4.9)	4.1(3.6-5.0)
Triglyceride, mmol/L	1.0(0.7-1.3)	1.2(0.8-1.4)	0.9(0.7-1.3)
High density lipoprotein, mmol/L	1.2(1.0-1.4)	1.1(0.9-1.4)	1.2(1.0-1.4)
Low density lipoprotein, mmol/L	2.4(1.9-3.1)	2.3(1.9-3.0)	2.4(1.9-3.2)
UA, μmol/L	314.0(241.5-383.0)	319.5(255.5-387.3)	340.0(257.5-411.5)
Glycated hemoglobin, mmol/L	6.0(5.6-6.7)	6.2(5.7-7.5)	5.9(5.6-6.5)
Homocysteine, μmol/L	12.0(10.1-15.2)	12.0(9.8-17.3)	12.0(10.3-14.9)

Data are presented as median (IQR) or number (%). Abbreviations: IQR, interquartile range; TIA, Transient cerebral ischemia; NIHSS, National Institute of Health stroke scale; sICH, symptomatic intracranial hemorrhage; UA, Uric Acid.

Supplement Table S2. The p-values of pairwise comparisons of AUCs on the testing set for different models with the Delong test

Model	RF	SVM	XGBoost
**LR**	<0.001	0.898	<0.001
**RFC**		<0.001	0.424
**SVM**			<0.001

The significant difference between AUCs is defined as p-value < 0.05. Abbreviations: AUC, the area under receiver operating characteristic curve; LR, logistic regression; RF, random forest; SVM, support vector machine; XGBoost, extreme gradient boosting
